# Human Rabies in China, 1960-2014: A Descriptive Epidemiological Study

**DOI:** 10.1371/journal.pntd.0004874

**Published:** 2016-08-08

**Authors:** Hang Zhou, Sirenda Vong, Kai Liu, Yu Li, Di Mu, Liping Wang, Wenwu Yin, Hongjie Yu

**Affiliations:** 1 Division of Infectious Diseases, Key Laboratory of Surveillance and Early-warning on Infectious Disease, Chinese Center for Disease Control and Prevention, Beijing, China; 2 Torch High Technology Industry Development Center, Ministry of Science and Technology, Beijing, China; 3 World Health Organization, China Office, Beijing, China; 4 Division of Infectious Diseases, Xiaogan Center for Disease Control and prevention, Xiaogan City, China; 5 School of Public Health, Fudan University, Key Laboratory of Public Health Safety, Ministry of Education, Shanghai, China; Wistar Institute, UNITED STATES

## Abstract

**Background:**

Rabies in China remains a public health problem. In 2014, nearly one thousand rabies-related deaths were reported while rabies geographic distribution has expanded for the recent years. This report used surveillance data to describe the epidemiological characteristics of human rabies in China including determining high-risk areas and seasonality to support national rabies prevention and control activities.

**Methods:**

We analyzed the incidence and distribution of human rabies cases in mainland China using notifiable surveillance data from 1960–2014, which includes a detailed analysis of the recent years from 2004 to 2014.

**Results:**

From 1960 to 2014, 120,913 human rabies cases were reported in mainland China. The highest number was recorded in 1981(0.7/100,000; 7037 cases), and in 2007(0.3/100,000; 3300 cases). A clear seasonal pattern has been observed with a peak in August (11.0% of total cases), Human rabies cases were reported in all provinces with a yearly average of 2198 from 1960 to 2014 in China, while the east and south regions were more seriously affected compared with other regions. From2004 to 2014, although the number of cases decreased by 65.2% since 2004 from 2651 to 924 cases, reported areas has paradoxically expanded from 162 prefectures to 200 prefectures and from southern to the central and northern provinces of China. Farmers accounted most of the cases (65.0%); 50–59 age group accounted for the highest proportion (20.5%), and cases are predominantly males with a male-to-female ratio of 2.4:1 on average.

**Conclusions:**

Despite the overall steady decline of cases since the peak in 2007, the occurrence of cases in new areas and the spread trend were obvious in China in recent years. Further investigations and efforts are warranted in the areas have high rabies incidence to control rabies by interrupting transmission from dogs to humans and in the dog population. Furthermore, elimination of rabies should be eventually the ultimate goal for China.

## Author Summary

China is a high-risk environment for rabies, with human rabies cases second only to India globally. This paper reviews 55 years of rabies epidemiology in mainland China, and detailed analysis of data in recent years. In this study, notifiable surveillance data were analyzed and found that rabies still remains a serious public health problem in China, the east and south regions were more seriously affected compared with other regions, however, the occurrence of cases in new areas and the spread trend were obvious in China in recent years. Moreover, males in rural areas had higher risk of infection than residents in urban areas, a clear seasonal pattern has been observed with a peak in August. These findings indicated a clear need to increase government and public consciousness with regard to the potential risk of rabies and the means of avoiding the disease. Further efforts should be strengthen specially in the high spot areas.

## Introduction

Rabies is a zoonotic disease caused by viruses of genus *Lyssavirus*, it’s also a vaccine-preventable viral disease which occurs in more than 150 countries and territories. Although a number of carnivore and bat species serve as natural reservoirs, worldwide rabies in dogs is the source more than 95% of human infections [1–3]. A recent study estimates that globally canine rabies causes approximately 59,000 (95% Confidence Intervals: 25–159,000) human deaths [4], As WHO reported, most cases occur in Asia and Africa [5,6]. In Asia, rabies has been controlled or eliminated for decades in Malaysia, Japan and many island countries or regions [7]. However, India is reported to have the highest incidence of rabies globally [6,8], and China is a high-risk environment for rabies, with human rabies cases second only to India [7,9]. Here we describe the rabies surveillance system and characterize the epidemiology of the disease in China. We focused on the recent years, to identify high-risk areas and their specificities to help plan resource allocation for rabies interventions.

## Methods

### The Chinese Notifiable Disease Reporting System

The Chinese Notifiable Disease Reporting System (NDRS) which was initiated in 1950s is the fundamental communicable disease surveillance system in China [10]. In the 1950s to the middle of 1980s, case numbers of communicable diseases in counties were monthly reported by mail through municipal and provincial health agencies until they finally reach the Ministry of Health taking a rather long time of 30 to 40 days. From the middle of 1980s to the end of 2003, although computers started to be used in some areas to collect and aggregate data of infectious disease, the whole country’s data still had to go through all the levels to reach the MOH which caused a time lag in reporting, making it hard for the early detection of outbreaks. After SARS outbreak in 2003, the Chinese government strengthened the construction of public health information system. On January 1st, 2004, the Real-time Notifiable Infectious Disease Reporting System was put into use nationwide, realizing the timely online monitoring of individual cases which marks a leap in the surveillance of communicable diseases in China.

### Case definitions

WHO classified human rabies cases to suspected, probable and confirmed cases. A ***suspected rabies case-patient*** was defined as a case that is compatible with a clinical case definition; a ***probable rabies case-patient*** was defined as a suspected case plus a reliable history of contact with a suspected rabid animal; a ***confirmed case-patient*** was defined as a suspected or probable case that is laboratory-confirmed [6].

In China, from beginning, Rabies cases were diagnosed according to the unified diagnostic criteria issued by Chinese Ministry of Health, this diagnostic criteria was modified in 2008.

Before modification, a ***probable rabies case-patient*** was defined as a patient licked, bit or scratched by dog, cat or other mammals with clinical symptoms of prickling or itching sensation at the site of bite, progressing within days to agitation, anxiety, confusion, hydrophobia, aerophobia, and paralysis of muscles or cranial nerves. A ***confirmed case-patient*** was defined as a probable rabies patient with laboratory evidence of rabies virus infection detected by direct fluorescent antibody test (DFA), or by virus isolation testing of clinical specimens.

After modification, clinical symptoms of case-patient were classified into two forms according to the case definition of World Health Organization (WHO), furious rabies and paralytic rabies. Symptoms of furious rabies were similar to the definition before modified, while paralytic rabies without exhibit signs of hyperactivity or hydrophobia, starting at the site of the bite or scratch, muscles gradually become paralyzed, progressing with the systemic flaccid paralysis. With epidemiological history, patient either of furious rabies or paralytic rabies was defined as a probable case-patient. A confirmed case-patient was defined as a probable rabies patient with laboratory evidence of rabies virus infection detected by direct fluorescent antibody test (DFA), reverse-transcriptase polymerase chain reaction (RT-PCR), or by virus isolation testing of clinical specimens.

### National surveillance for rabies

Rabies was on the initial list of notifiable diseases in mainland China from 1950s. All probable rabies cases and those laboratory-confirmed were required to be mandatory reported when those patients sought medical consultations.

Appropriate clinical specimens, including saliva, cerebrospinal fluid (CSF), urine, nuchal skin biopsies, or brain tissues post mortem, were collected upon the patient’s syndromes by nurses and shipped immediately for virus testing following standardized procedures. Specimens were sent to the local or provincial level CDCs and there they were tested for rabies virus by DFA, RT-PCR, or virus isolation using the protocols and kits released by the China CDC. Only very small portion of specimens were sent to the China CDC tested by virus isolation for difficulty in testing in province CDC or further characterization of the results.

### Data collection

From 1950 to 2003, number of cases and deaths by province were reported monthly to the China CDC, age and sex aggregate data was added from 1988.

After 2004, each probable and confirmed case-patient with individual data was required to be reported from all health care facilities nationwide to the China CDC by clinicians within 24 hours after diagnosis using a standardized form to collect data about demographic information (gender, date of birth, and location), residence type (rural or urban), case classification (probable or confirmed case), date of onset and, if applicable, date of death; all data were reported electronically online to the China CDC.

### Data analysis

Descriptive statistics included frequency analysis for categorical variables, means and standard deviations for normal distributions, or medians and inter quartile ranges (IQR) for continuous variables.

As the quality of data from 1950 to 1959 was not very stable, we calculated temporal trends of cases and deaths from 1960 to 2014, geographic distribution of total cases was shown and patterns of seasonal distribution, sex, age and occupation was counted. Annual population denominators during the study period were obtained from National Bureau of Statistics of China.

Statistical analysis was performed using SPSS (v17.0, SPSS Inc, Chicago, IL, USA). *ArcGIS* 10.0 (ESRI, Redlands, CA, USA) was used to assess the geographical distribution of cases across all the provinces in mainland China. For all analyses, probabilities were 2-tailed and a p-value of <0.05 was considered statistically significant.

### Ethical approval

It was determined by the National Health and Family Planning Commission, China, that the collection of data from human rabies cases was part of a continuing public health surveillance of a notifiable infectious and was exempt from institutional review board assessment.

## Results

### Overall epidemic trend

**Fig 1:**
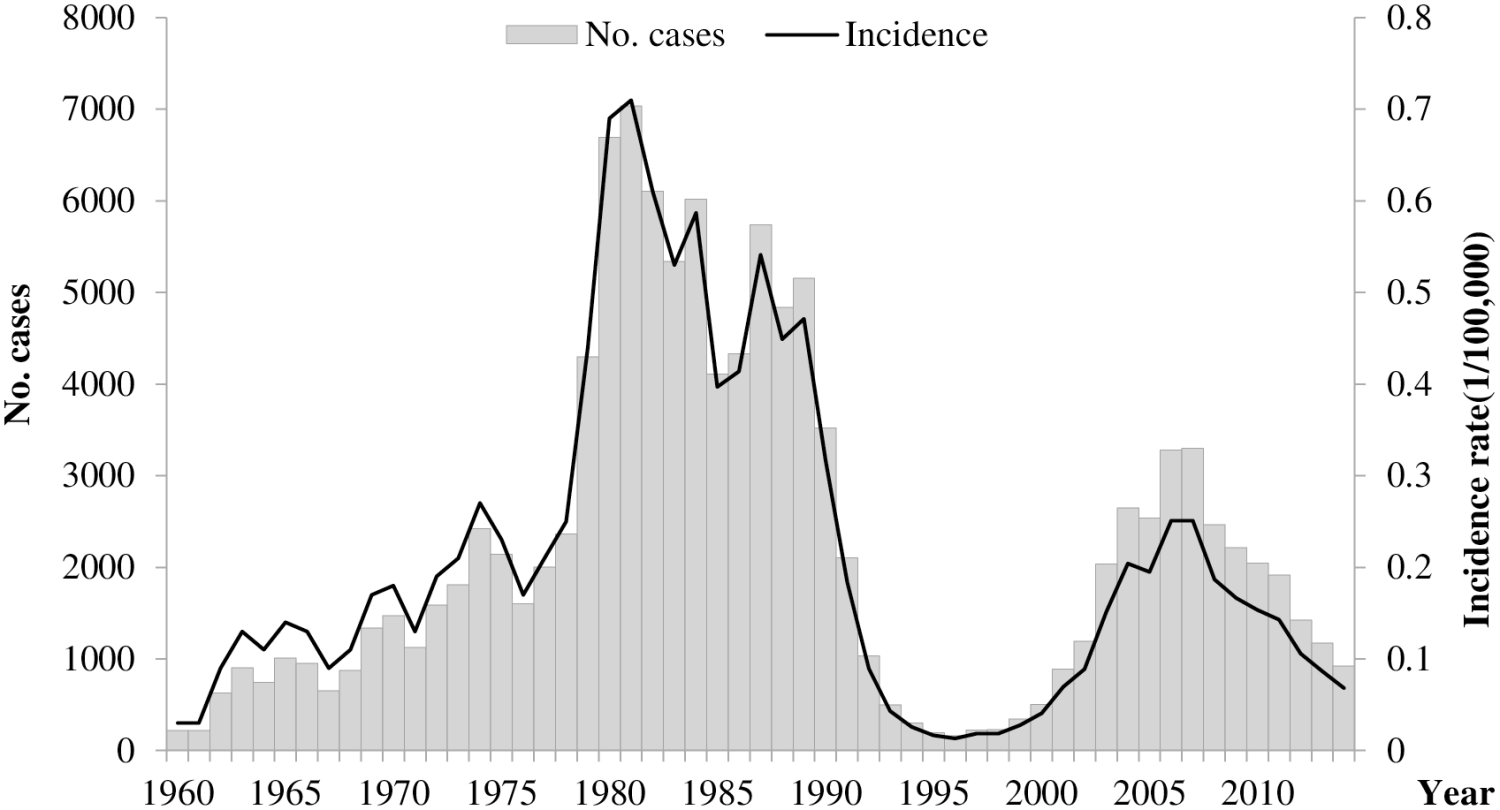
The incidence and number of human rabies cases reported in mainland China, 1960–2014 (N = 120,913).

From 1960 to 2014, China reported 120,913 human rabies cases with a yearly average of 2198, including 23,932 (19.8%) cases were reported during 2004–2014 (Fig 1). The highest incidence was observed in 1981(0.71/100,000, 7037 cases). When comprehensive control measures were conducted, such as management of stray dogs, vaccination of dogs, PEP of the exposures, the incidence rate decreased from the 1980s to the 90s and reached the lowest point in 1996 (0.01/100,000, 159 cases). However, the incidence rate increased again in 1997 to reach a second peak in 2007(0.25/100,000, 3300 cases). Since then the incidence rate has gradually decreased to reach 0.07/100,000 in 2014 (n = 924 cases).

### Geographical distribution

Historically—during 1960–2014 –all provinces reported human rabies cases were reported in all provinces with a predominance in the eastern and southern regions. Before 1996 (the lowest reporting year; n = 159 cases), human rabies were mainly concentrated in southern, central and northeast of China, while the northeast area maintained at a relatively low reporting level.

From 2004 to 2014, cases were mainly prevalent in southern regions, namely in Guangxi, Guizhou, Guangdong and Hunan provinces which accounted for 52% of the total cases. However, cases in these provinces decreased after 2007, while in the north, rabies cases still increased in recent years, such as Shanxi, Inner Mongolia and Beijing by 4800%, 600% and 600%. For Yunnan and Shaanxi, increased in 2011 and decreased in 2014.

**Fig 2:**
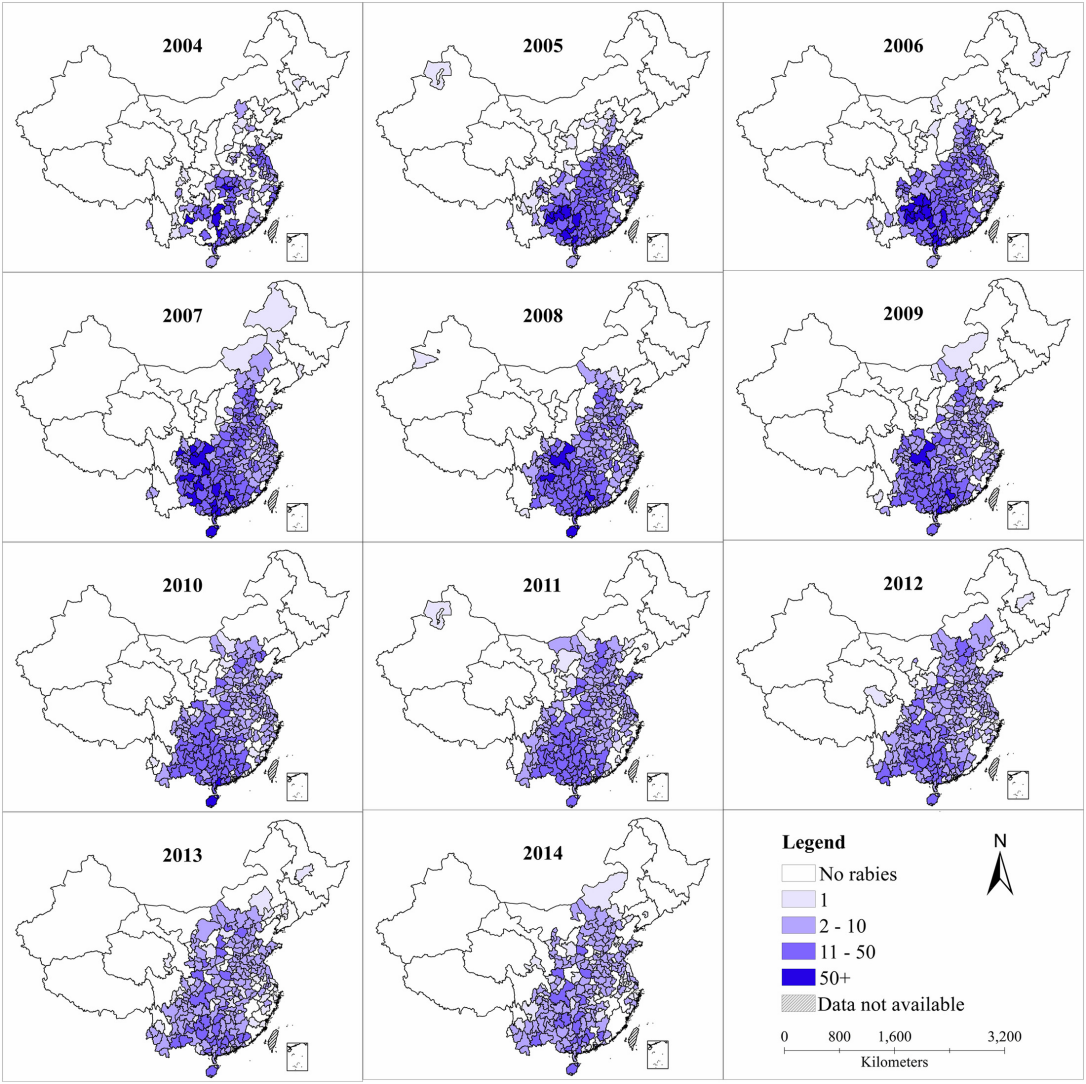
Spatiotemporal distribution of human rabies by prefecture level in mainland China, from 2004 to 2014.

In 2004, 2,651 cases were reported in 162 prefectures mainly in the eastern and southern regions while 924 (-65.2%) cases were reported in 200 (+23.5%) prefectures in 2014 (Prefecture is administrative subdivision of provincial-level division, which is a level between the provincial and the county level in China). Although the number of cases decreased in the past recent years, the number of reported areas expanded paradoxically with a spread into the northern and western regions of China. For example, Shanxi province had only had one prefecture that reported only one case in 2004 while—in 2014 –there were 8 prefectures that reported cases (n = 43). Hebei has 4 prefectures reported 7 cases in 2004 while 10 prefectures reported 56 cases. In the northeast region and northwest region, the number of reported prefectures were both very low, but Shaanxi had a large rise after 2008, no case were reported in 2008 and 3 prefectures reported 20 cases in 2014 and reported 26 cases in Hanzhong and Shangluo prefectures peaking in 2009. However, south and central regions still remained high rabies incidence for a number of years (Fig 2).

**Fig 3:**
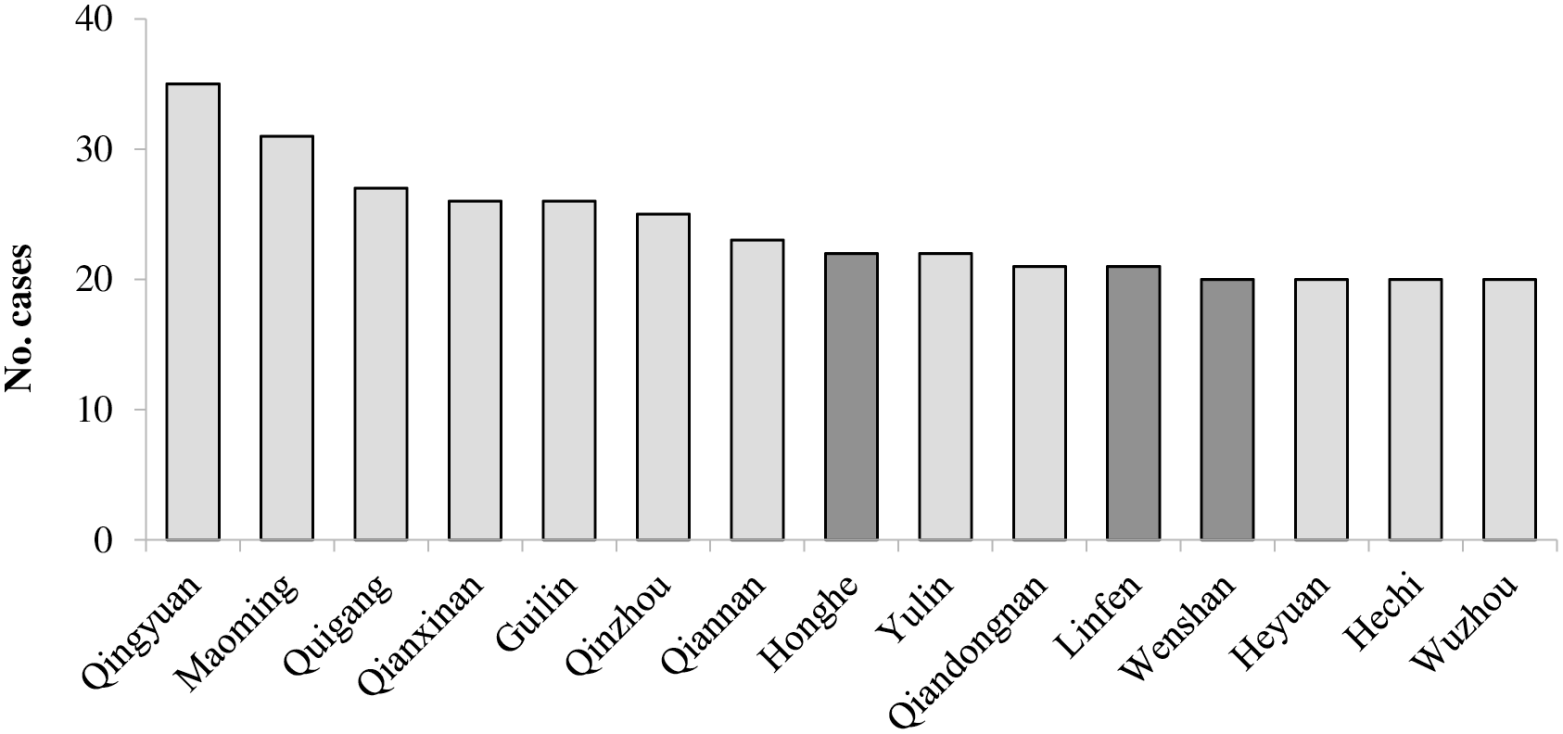
The variation of average cases in high risk prefectures from 2010 to 2014 compared with 2004 to 2009. Note: The dark gray column means the average cases during 2010 to 2014 were increase than 2004 to 2009, the light gray column means cases were decrease than before.

When focusing on prefectures with relatively high number of reported cases—during 2010–2014 –we identified 15 prefectures that accumulatively reported more than 100 cases (Fig 3). 15 prefectures were located in 5 provinces, Guangxi (6 prefectures), Guizhou and Guangdong (3 prefectures each), Yunnan (2 prefectures) and Shanxi (1 prefecture). These provinces were all concentrated in southern and central areas of China. The top 3 prefectures were Qingyuan, Maoming and Guigang. All these provinces and prefectures were in southern China. In addition, of these 15 prefectures, the number of onset decreased in 12 by 53.85% on average when comparing 2004–2009 with that of 2010–2014 (highest 70.1% in Qianxinan, in Guizhou province). In contrast cases increased in Honghe (average of 13 cases, 144.4%) and Wenshan (21 cases, 122%), Yunnan province, and in Linfen (21 cases, 2100%) from Shanxi province.

### Seasonal pattern

**Fig 4:**
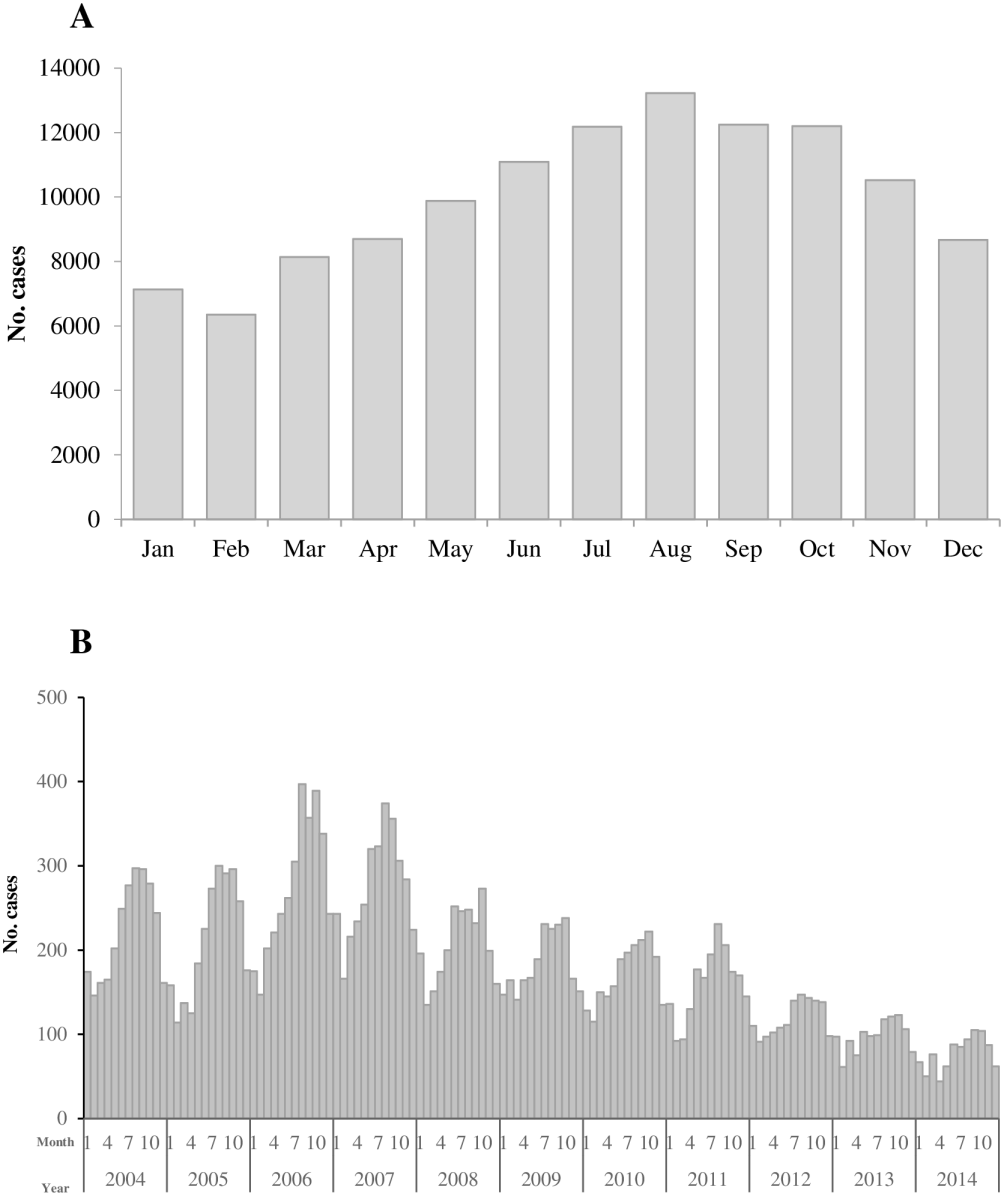
The reported human rabies cases by month in mainland China. (A) The reported human rabies cases by month in mainland China from 1960 to 2014. (B) The reported human rabies cases by month in mainland China from 2004 to 2014.

Since 1960, Cases occurred throughout the year; however, most cases (59% of total cases) were reported from June to November and the numbers peaked usually in August (11.0% of total cases) (Fig 4A). During these 55 years, August maintained the peak month in 29 years, followed by September (9 years) and October (8 years). Following the peak year of 2007, as the reported number of cases steadily decreased year by year the seasonal pattern was less apparent especially from 2012 to 2014 (Fig 4B). The range of cases in the highest and lowest month in 2007 and 2014 was 208 and 61, respectively.

### Demographic features

**Fig 5:**
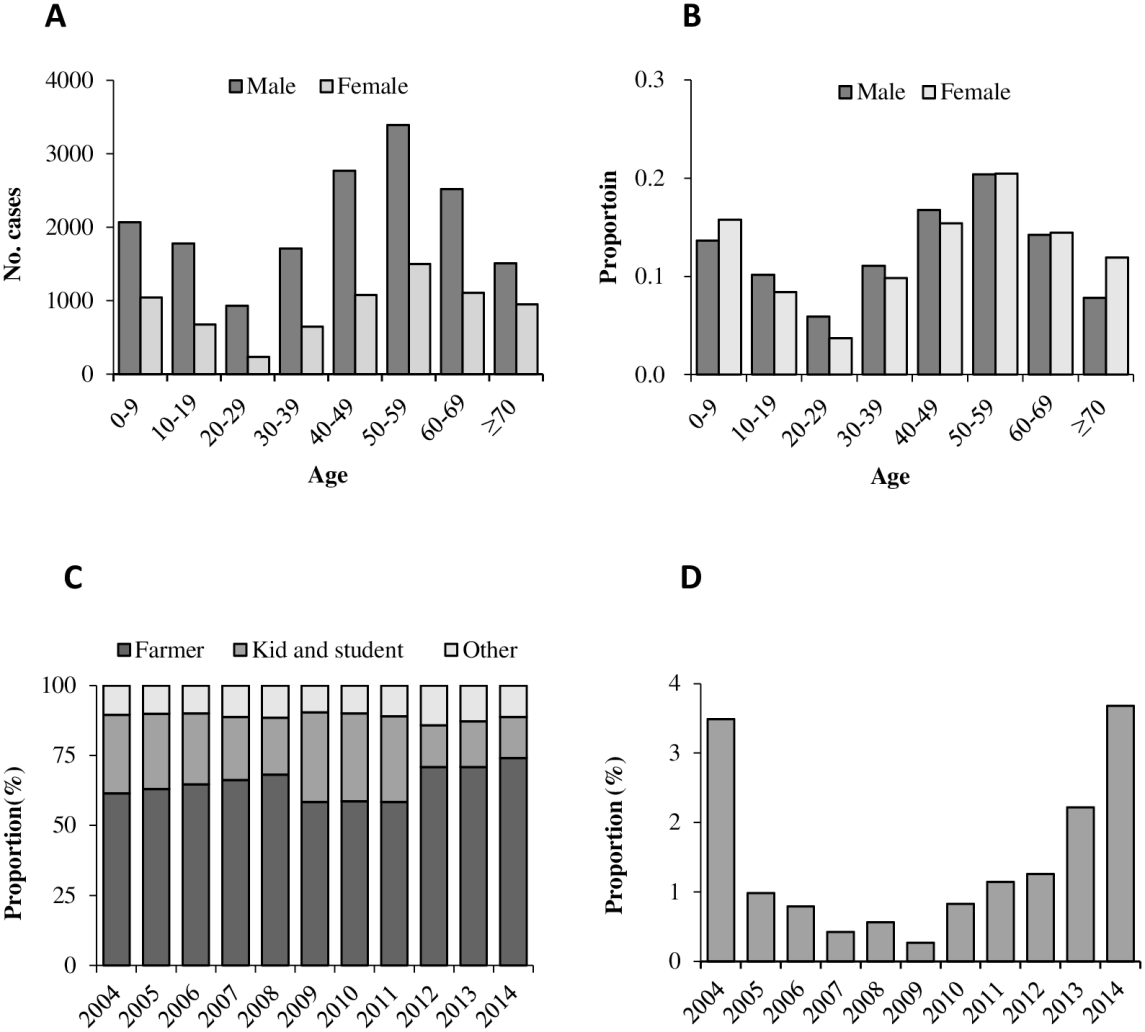
The proportion of gender and diagnosis of human rabies cases by age and year, 2004–2014. (A) The number of male and female of human rabies cases by age. (B) The proportion of male and female of cases by age. (C) The proportion of occupation of cases by year of illness onset. Kids includes kindergarten children and diaspora children, student includes primary, secondary and college students. Others included teacher, laborers, self-employed and unemployed, workers, food industry personnel, retired and cadres of staff, etc. (D) The proportion of confirmed cases by year of illness onset.

Demographic data has only been available since 1988. During 1988–2014, the overall male-to-female ratio of 2.4:1; this ratio was similar in different years (*P*>0.05). Overall, the median age was 46 years (IQR: 23–59); females were on average older than males (49 years vs 46 years of age). From 1988 to 2003, 0–9 age group reported the highest proportion of the total cases (24.4%), then followed by 10–19 age group (17.2%), and after 70yrs group (3.3%) accounted smallest proportion. However, from 2004 to 2014, 50–59 age group accounted for the highest proportion (20.5%), then followed by 40–49 age group (16.1%), while 20–29 age group (4.9%) had the lowest proportion as shown in Fig 5A and 5B. Farmers accounted for 65.0% of the total cases, and then followed by students and children (24.1%). This pattern has been consistent year by year since 1992 in China. (Fig 5C). Rural cases accounted for 78.7% of all cases since 2004, this proportion was lowest of 75.2% in 2004 and highest of 80.9% in 2005, no statistical difference between different years (P>0.05).

The proportion of lab-confirmed cases was very low in China, and average of 1.2% cases were tested from 2004 to 2014, however, this proportion increased in recent years (P<0.01) (Fig 5D). The median time of onset-to-death interval of rabies cases was 3 days, the median time of onset-to diagnosis interval was 2 days, the median of diagnosis-to-death interval was 1 day, we analyzed the three median by year, and the data maintained constant in every year from 2004.

## Discussion

China experienced a serious re-emergence from the disease, in the 1980s with an average of 5,500 cases reported annually [11,12]. There were two peaks of reported rabies case in 1981 and 2007, China has made considerable efforts to control the rabies epidemic in the 80’s and 90’s to reach a low level of incidence in the mid-90’s, such as management of stray dogs, vaccination of dogs, PEP of the exposures. however, the incidence rose again and peaked in 2007, when 3,300 cases were record, and human rabies was among the top three causes of human death due to infectious diseases in the country [13,14]. Since 2007 the government has recognized the urgency and invested both the national and local level to curb the re-emergence. For instance, human rabies vaccine is not included in the national immunization program in China, therefore, bitten subjects should pay for the PEP fees by themselves. However, in the high risk provinces, such as Guizhou, Guangxi and Guangdong, they added the PEP fees, especially the vaccine fees into social insurance system in recent years, higher percentage of bitten subjects will get PEP than observed in previous years. As a result, the incidence has declined steadily since the peak in 2007. In the high risk prefectures, cases in 80% high risk prefectures were decreased on average when comparing 2004–2009 with that of 2010–2014.

Despite major control achievements were conducted for the past decade, rabies is still an important public health problem in China with nearly 1,000 human rabies in 2014; only second to India in terms of burden of disease [15–18]. Moreover, although China has had a steady decline in the number of cases over past years, cases had paradoxically occurred in a wider geographical area, 162 prefectures were reported in 2004 while 200 prefectures in 2014, rabies expanded to northern and western areas in recent years, such as Shaanxi and Inner Mongolia. This tendency indicates that although the comprehensive measures were conducted, such as central and local government enhanced invest for establishing standard post-exposure clinics, reimbursement of vaccine fees in high risk areas, the source of rabies infection might have been spreading. The reason may due to the urbanization, convenient means for transportation helped the spread of rabies.

Compatible with another study [19], more cases were reported from June through November and usually peaked in August from the surveillance results since 2004. This may be related to relative higher exposure risk in these months and longer incubation period for rabies. Firstly, people wear less clothes from May to September in China, if they attacked by rabies animals in these months, wound would be more serious than winter. Secondly, the average incubations were relative longer than other acute infectious disease, such as influenza, dengue, after an average incubation period of two months, individuals may present symptoms. Therefore, November still maintained relatively high incidence of rabies.

The historical data analysis also showed an interesting finding. The 0–9 age group reported the highest proportion of the total cases before 2003, but the 50–59 age group accounted for the highest proportion from 2004 to 2014 which was contrast to other studies [20–25]. We can conclude that 0–9 age group had the largest number of rabies case before 2003 informing that the epidemic age group has changed in recent years. This might be due to family planning in China and per household income was higher than before. Now, the majority of families had only one child and parents are indulgent to their children. Parents wound bring their children for vaccination after dog bites as soon as possible. However, parents might don’t get vaccination after dog bites if the wound was not serious. The rate of vaccination after dog bites among children was higher than that among adult in recent years [26]. Furthermore, young adults in rural areas go to cities to earn money and then return to their hometown for the spring festival. As a result, the majority of residents of rural areas between the months of May and August are elderly people and children.

Nearly 80% cases occurred in rural areas of China since 2004, two main reasons may lead to this result, firstly, in most area of China, the costs of PEP didn’t be covered in the insurance system, therefore, some exposures didn’t go to seek PEP service due to the poor economic status. Secondly, the PEP clinics usually distributed in downtown or urban area and far from the rural area, the clinic was not convenient for the rural residents.

The proportion of lab-confirmed cases was still very low in China, there were two main reasons. The first reason is most rabies cases resided in rural areas, where were far from the county or prefecture level hospitals, however, the clinics in village usually couldn't collect samples for patients. Moreover, the median time of onset-to-death interval was very short for rabies cases (3 days), therefore, many cases were dead before were transferred to big hospitals to collected samples. The second reason is if the patient was suspected rabies, the patient may presented some offensive behaviors, this may result in doctors too hard to collect the saliva or CSF samples. Because majority of cases were clinically diagnosed, some cases may be underreported. The real number of rabies cases may be higher and epidemic area may be larger in China. The other finding is the major information gaps in surveillance of rabies. Surveillance was not sufficiently sensitive and reactive to characterize the cases and identify clusters of cases or hot-spots at the most peripheral level. Prompt analysis and feedback of the information with recommendations for investigations and control could help the local authorities to support rapid control intervention in dogs and prevention campaign in the affected areas. Therefore, China is planning to promote the rabies surveillance system, all of the cases will be investigated for exposure history, and every probable case will be required to collect samples for testing. Case information will be informed to agriculture department so that the vaccination and control measure for suspected animal could be immediately conducted.

In summary, rabies was endemic in China and all provinces reported cases. But the prevalence of human rabies in Guangxi, Guizhou, Hunan, Guangdong was more serious. From 2010 to 2014, high risk prefectures were mainly concentrated in southern and central areas of China, however, cases in 80% high risk prefectures were decreased on average when comparing 2004–2009 with that of 2010–2014. As shown in other studies, the geographic cluster might be not relative to lineage of rabies virus because 80% of Chinese rabies strains belonged to the same lineage [27,28]. Frequency of exposure to dog bite, dog immunization rate, and post-exposure prophylaxis were associated with incidence rate of rabies.

Although rabies is a horrifying, fatal, and incurable disease, it is 100% preventable, and it’s still not properly addressed by the government. Comprehensive control measures including political commitment to control programs, inter-sectorial coordination, sensitive surveillance systems, accessibility to modern rabies vaccine, awareness education and cooperation should be strengthened to eliminate rabies in China [29]. Of these measures, the administration of raising dogs and improvement of dog immunization are of most importance. Fortunately, The State Council outlined their future plan in the document Long-term Animal Disease Prevention and Control Plan (2012–2020), which lists the rabies as one of 16 domesticated animal diseases to be prioritized for prevented and controlled. Moreover, further investigations and efforts are warranted in the high spot area to control rabies by interrupting transmission from dogs to humans and in the dog population.

### Conclusions

Rabies is notifiable in animals and humans in China, despite the overall steady decline of cases since the peak in 2007, the occurrence of cases in new areas and the spread trend were obvious in China in recent years. Further investigations and efforts are warranted in the high spot area to control rabies by interrupting transmission from dogs to humans and in the dog population. Elimination of rabies should be eventually the ultimate goal for China.

## Supporting Information

S1 Checklist STROBE Checklist. (DOC)
